# Analysis of MHC class II-bound CyHV-2 peptides in *Carassius gibelio* using mass spectrometry

**DOI:** 10.1128/jvi.01870-25

**Published:** 2025-12-29

**Authors:** Chen Xu, Fangxing Yu, Jiajia Ye, Mingyang Xue, Zhenyu Huang, Nan Jiang, Yan Meng, Yuding Fan, Weiguang Kong, Ya Zheng, Yong Zhou

**Affiliations:** 1 Yangtze River Fisheries Research Institute, Chinese Academy of Fishery Sciences 499154 , Wuhan, China; 2 Shanghai Normal University, College of Life Sciences 12544, Shanghai, China; 3 Key Laboratory of Breeding Biotechnology and Sustainable Aquaculture, Institute of Hydrobiology, Chinese Academy of Sciences 53041 , Wuhan, China; Fred Hutchinson Cancer Center Vaccine and Infectious Disease Division, Seattle, Washington, USA

**Keywords:** teleost, polymorphism, immunopeptidome, MHC-II peptides, *Cyprinid herpesvirus *2

## Abstract

Class II major histocompatibility complexes (MHC-II) are a highly polymorphic and multigenic family of molecules that present exogenous peptides to CD4^+^ helper T cells, thereby activating the host adaptive immune system. In this study, we systematically analyzed the genomic distribution and tissue-specific expression of MHC-II genes in *Carassius gibelio*. The *Cagi*-DDA/DFA molecule was found to be highly expressed in the spleen and head kidney, moderately expressed in the intestine, gills, and trunk kidney, and expressed at low levels in the liver and brain. A polyclonal antibody was generated against the most prevalent *Cagi*-DDA/DFA allele in the population. Using immunopeptidomics, we identified viral peptides bound to *Cagi*-DDA/DFA molecules in the head kidney tissues of *C. gibelio* following *Cyprinid herpesvirus* 2 (CyHV-2) infection. A total of 276 antigen peptides were identified, originating from 39 viral proteins. Notably, viral proteins with high abundance and early expression profiles, such as ORF88, ORF121, and ORF141 proteins, were more likely to generate antigen peptides. The identified CyHV-2 peptide epitopes presented by *C. gibelio* MHC-II molecules provide candidate antigens required for anti-CyHV-2 vaccine development.

**IMPORTANCE:**

Vaccination represents a cornerstone in the prevention of infectious diseases, achieving substantial success in disease control. Upon immunization, protein-derived peptides are processed and presented by major histocompatibility complex class II (MHC-II) molecules, activating CD4^+^ T cells and triggering adaptive immune responses. *Cyprinid herpesvirus* 2 (CyHV-2), a pathogenic virus in crucian carp, poses a serious threat to global aquaculture. However, the absence of a comprehensive antigenic profile for CyHV-2 has hindered the development of effective vaccines. Here, we employed immunoaffinity purification coupled with mass spectrometry to systematically identify CyHV-2-derived peptides presented by MHC-II in *Carassius gibelio*. We identified 276 antigenic peptides originating from 39 viral proteins, which collectively delineate the antigenic landscape of CyHV-2 and provide a rational basis for the design of a vaccine against CyHV-2.

## INTRODUCTION

Peptides presented by major histocompatibility complex (MHC) molecules represent potential antigenic epitopes of a specific protein (1). CD4^+^ helper T cells recognize peptides bound to membrane-associated MHC class II (MHC-II) molecules and become activated, subsequently promoting the maturation and differentiation of CD8^+^ T cells and B cells (2–4). Therefore, precise identification of MHC-II-bound peptide motifs, including those derived from viral proteins, is essential for elucidating protein immunogenicity and developing effective immunoprophylactic strategies. *Cyprinid herpesvirus* 2 (CyHV-2, also known as *Cyvirus cyprinidallo* 2) is a linear double-stranded DNA virus that belongs to the genus *Cyprinivirus*, family *Alloherpesviridae* (5). CyHV-2 is the main pathogen responsible for the development of herpesviral hematopoietic necrosis disease, which can lead to a high fatality rate and large economic loss within the farmed gibel carp (*Carassius gibelio*) industry (6). To date, multiple CyHV-2 strains have been isolated from diseased fish in various countries and regions, and several complete genome sequences have revealed approximately 150 genes, most supported by transcriptomic or proteomic evidence (7–10). However, the repertoire of CyHV-2 peptides presented by MHC-II molecules remains unexplored, limiting our understanding of the viral protein immunogenicity.

Several methodologies have been developed for the identification of MHC-II-bound peptides, including immunoaffinity enrichment of MHC complexes, mild acid elution, and yeast display (11–13). Furthermore, bioinformatic tools have been introduced to predict and prioritize MHC-II-bound peptides (14, 15). Among these approaches, mass spectrometry (MS) combined with immunoaffinity purification has emerged as a powerful strategy for profiling the immunopeptidome (16, 17). Following MHC complex enrichment from cultured cells or tissue samples, numerous antigenic peptides can be identified in a single MS-based experiment (18). This method has been widely applied across various species and tissue types, with the notable exception of bony fish (19, 20). Therefore, it is necessary to elucidate the binding motifs of CyHV-2-derived peptides associated with MHC-II molecules in gibel carp.

The selection of appropriate antibodies is critical for the enrichment of MHC-II complexes and the subsequent identification of bound peptides (2). MHC-II molecules are heterodimeric proteins composed of α and β chains, featuring an open-ended peptide-binding groove that accommodates a conserved nine-residue core flanked by variable-length extensions at both termini (21, 22). In vertebrates, both the MHC-IIα and MHC-IIβ chains exhibit pronounced multigenicity and extensive polymorphism (21, 23, 24). In humans, MHC-II molecules are classified into HLA-DR, HLA-DP, and HLA-DQ groups, all located on the short arm of chromosome 6 with evident genetic linkage. Numerous monoclonal antibodies (mAbs) have been developed with specificities ranging from human MHC-II molecule classes and families to individual alleles (2). In teleosts, however, MHC-II genes are dispersed across distinct chromosomes without genetic linkage (21). Based on sequence characteristics, they are categorized into DA, DB, and DE groups (25). Among them, DA group genes possess conserved peptide-binding sites and high polymorphism, representing the classical MHC-II molecules in teleosts, whereas DB and DE groups display low polymorphism and lack conserved binding motifs (25). Although MHC tertiary structures are evolutionarily conserved, the amino acid sequences exhibit substantial divergence, rendering mammalian-derived mAbs generally ineffective for teleost MHC-II detection (21, 26). Consequently, there remains a lack of commercially available mAbs suitable for immunoaffinity capture of teleost MHC-II-peptide complexes.

In this study, we analyzed the tissue-specific distribution and expression patterns of various MHC-II molecules in gibel carp, identified the predominantly expressed MHC-II allele, and generated polyclonal antibodies (pAbs) against it. The antibody was validated for its suitability in enriching MHC-II complexes in gibel carp and was subsequently used to identify CyHV-2-derived antigen peptides. The results revealed that viral proteins with high abundance and turnover rates, such as ORF88, ORF121, and ORF141, were more likely to generate MHC-II-bound peptides. Collectively, our findings provide the first characterization of CyHV-2-derived antigenic epitopes presented by MHC-II molecules in gibel carp, offering valuable insights for the development of effective vaccines against CyHV-2 infection.

## MATERIALS AND METHODS

### MHC-II gene nomenclature

The reference genome of *C. gibelio* used in this study corresponds to GenBank accession number GCA_023724105.1. The nomenclature for *C. gibelio* MHC-II genes (*Cagi-dxa*/*Cagi-dxb*) follows the standardized guidelines established for all vertebrate species (27). The prefix “*Cagi*” is derived from the first two letters of the genus (*Carassius*) and species (*gibelio*). The letter “d” denotes class II, while the subsequent variable letter “x” indicates the specific locus, including DA, DB, and DE groups. The final letter “a” or “b” designates the MHC-II α- or β-chain, respectively.

### Cloning and sequencing of *Cagi*-DDA/DFA allelic variants

Total RNA was extracted from spleen tissues of 76 individual *C. gibelio* specimens bred at the Heilongjiang River Fisheries Research Institute, Chinese Academy of Fishery Sciences, using the FastPure Cell/Tissue Total RNA Isolation Kit (Vazyme, China). First-strand cDNA was synthesized with the HiScript III 1st Strand cDNA Synthesis Kit (Vazyme, China) following the manufacturer’s instructions. Full-length *Cagi-*DFA sequences were amplified by PCR using cDNA from all 76 individuals as templates. The primers used were: *Cagi*-DDA/DFA-up: CATCGACAAACATGGAGCTGT; *Cagi*-DDA/DFA-down: AGAATCCTGCAGAAACGGTC. PCR products were cloned into the pTOPO Univer Cloning Vector (Vazyme, China) and transformed into *Escherichia coli* competent cells. For each individual, at least three recombinant clones were selected for sequencing.

### Plasmid construction and detection of pAb specificity

The full-length coding sequences of various *C. gibelio* MHC-II genes were synthesized and subcloned into the pcDNA3.1 vector at the *BamHI* and *XhoI* restriction sites to generate MHC-II-Flag recombinant plasmids. Endotoxin-free plasmids (10 μg) were transfected into GiCB cells using a Takara transfection kit (Takara, Japan) according to the manufacturer’s instructions. Transfected cells were cultured in Hyclone Medium 199 (Hyclone, USA) supplemented with 10% fetal bovine serum at 28°C for 48 h. Western blot analysis was performed to examine the recognition specificity of the home-made anti-*Cagi*-DDA/DFA pAb toward different *C. gibelio* MHC-II molecules. A rabbit-derived anti-Flag pAb (Abcam, UK) was used to verify the expression of various *C. gibelio* MHC-II proteins in GiCB cells.

### Protein extraction

Head kidneys were collected from *C. gibelio* (mean weight: 150 ± 30 g) infected with CyHV-2 and washed with PBS at least three times to remove residual blood. Tissues were resuspended in lysis buffer containing 20 mM Tris-HCl (pH 7.5), 150 mM NaCl, 0.1% DDM, and 1 × protease and phosphatase inhibitor cocktail (Roche, Switzerland), followed by homogenization and sonication for 5 min on ice. Lysates were centrifuged at 12,000 × *g* for 10 min at 4°C, and the supernatants were collected for protein concentration determination and MHC-II complex immuno-enrichment. For membrane protein extraction from various tissues, brain, intestine, gill, liver, spleen, head kidney, and trunk kidney were collected from five healthy *C. gibelio*, and membrane proteins were isolated using the Mem-PER Plus Kit (Thermo Scientific, USA) according to the manufacturer’s instructions.

### Western blot

A total of 30 μg of protein from various *C. gibelio* tissues was mixed with SDS-PAGE loading buffer and denatured by heating at 95°C for 10 min. Proteins were separated on a 12% SDS-PAGE gel and transferred to polyvinylidene fluoride membranes using a wet transfer system at 100 V for 50 min. Membranes were blocked with 5% skim milk in TBST for 2 h at room temperature (RT), followed by overnight incubation at 4°C with 1 μg of home-made rabbit-derived anti-*Cagi*-DDA/DFA polyclonal antibody diluted in TBST containing 1% skim milk. After five washes with TBST, membranes were incubated with horseradish peroxidase-conjugated goat anti-rabbit IgG for 2 h at RT, followed by five additional TBST washes. Protein signals were visualized using the Western Lightning ECL substrate system (Perkin Elmer, USA) and imaged with the ChemiDoc XRS+ system (Bio-Rad, USA).

### Immunoprecipitation of *C. gibelio* MHC-II complexes

Approximately 10 mL of head kidney lysate containing ~10 mg total protein was aliquoted into five 2 mL Eppendorf tubes for MHC-II complex enrichment. The enrichment protocol was adapted from the method described by Purcell et al. (2). Briefly, 1 mg of rabbit-derived anti-*Cagi*-DDA/DFA pAb was covalently cross-linked to 1 mL of pre-washed settled Protein A magnetic beads (GenScript, USA) using 20 mM dimethyl pimelimidate dihydrochloride for 30 min at RT. The reaction was quenched with 50 mM Tris (pH 7.5) for 15 min at RT. The antibody-conjugated beads were collected using a magnetic separator and washed five times with wash buffer (20 mM Na_2_HPO_4_ and 0.15 M NaCl, pH 7.0). Subsequently, the beads were incubated with the head kidney lysates on a rotator at RT for 2 h to capture MHC-II immune complexes. Beads were then washed five times with PBS, and bound complexes were eluted with 10% acetic acid. The eluted samples were transferred into 10 kDa ultrafiltration devices and centrifuged at 3,000 × *g* to remove MHC-IIα and MHC-IIβ chains. The flow-through was dried under low temperature and stored at –20°C until further use.

### Fractionation of MHC-II peptidome and LC-MS/MS analysis

All peptides were fractionated using C18 columns (Agilent Technologies, USA) under high-pH reversed-phase conditions, as previously described (28). Briefly, peptides were dissolved in 25 mM ammonium formate (NH_4_FA, pH 10), loaded onto C18 columns pre-equilibrated with the same buffer, and sequentially eluted with increasing concentrations of acetonitrile (ACN; 5%, 10%, 20%, 30%, 40%, and 75%) in 25 mM NH_4_FA (pH 10). All fractions were lyophilized, resuspended in 0.1% formic acid, and subjected to LC-MS/MS analysis using a Q-Exactive Plus mass spectrometer (Thermo Scientific) coupled with an EASY-nLC 1200 system.

Peptides were separated on a C18 nano-LC analytical column (Acclaim PepMap C18, 75 μm × 25 cm, 2 μm, 100 Å, Thermo Scientific) at a flow rate of 300 nL/min with a 120-min linear gradient from 5% to 80% solvent (0.1% formic acid in 100% ACN). The spray voltage was set to 2.2 kV. Data-dependent acquisition (DDA) was performed with the following settings: full MS scan range of 300–1,800 *m/z*, AGC target of 5 × 10^5^, maximum injection time of 120 ms, normalized HCD collision energy of 28%, 20 MS/MS scans per cycle, precursor charge states of 2–4^+^, MS/MS resolution of 35,000, and a dynamic exclusion duration of 15 s.

### LC-MS/MS data interpretation

Open search analysis of the MHC-II peptidome was performed using the pFind 3.2 search engine (29). RAW files were searched against the *C. gibelio* and CyHV-2 YZ-01 strain proteome databases. The search parameters were set as follows: no enzyme specificity; precursor (MS1) mass tolerance of 20 ppm; fragment (MS2) mass tolerance of 0.02 Da; a false discovery rate of 1% at both peptide and protein levels; methionine oxidation and protein N-terminal acetylation were set as variable modifications. Proteome Discoverer 2.4 (Thermo Scientific) was used for the analysis of membrane proteomics data. As the membrane proteins were digested with trypsin prior to MS analysis, database search parameters were set accordingly, as described in our previous publication (30).

## RESULTS

### Distribution and expression characteristics of *C. gibelio* MHC-II molecules

**Fig 1:**
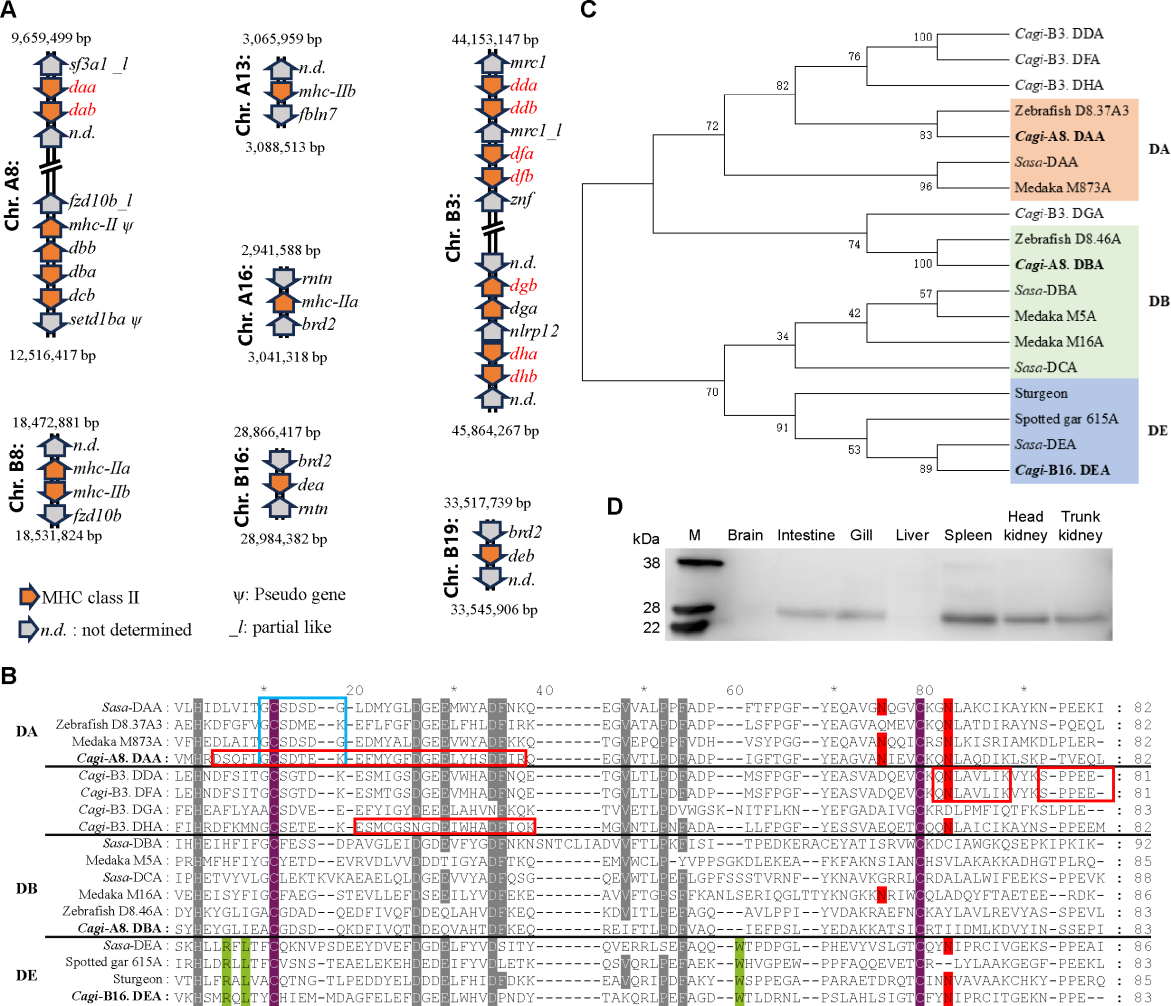
Distribution and expression of *C. gibelio* MHC-II molecules. (**A**) Gene map of *C. gibelio* MHC-II reference sequences. Genes are arranged according to their chromosomal positions (not to scale). Red indicates MHC-II molecules identified by MS. (**B**) Multiple sequence alignment of α1 domain sequences in MHC-II molecules. Purple: cysteine residues predicted to form disulfide bridges; Red: peptide-backbone interacting residues; Lime green: DE group specific residues; and Gray: highly conserved residues. Blue frame highlights DAA-lineage-specific or similar motifs; Red frame indicates peptides identified by MS. (**C**) Phylogenetic tree analysis of MHC-II molecules from *C. gibelio* and other representative teleost species (25). (**D**) Western blot validation of *Cagi*-DDA/DFA expression in membrane protein fractions from multiple *C. gibelio* tissues.

A total of 20 MHC-II genes, including both MHC-IIα and MHC-IIβ, were annotated in the *C. gibelio* genome. These genes were distributed across seven distinct chromosomes, including chromosomes A8, B3, and B8 (Fig. 1A). They encompassed the known teleost MHC-II groups DA, DB, and DE, as well as several undefined types, which were designated based on their sequence characteristics and chromosomal locations (Fig. 1B and C). For example, the classical DA group, comprising the DAA and DAB molecules, was located on chromosome A8, whereas chromosome B3 contained eight MHC-II genes—the largest number among all chromosomes (Fig. 1A). Phylogenetic analysis revealed that the MHC-II genes on chromosome B3 were evolutionarily close to the classical DA group (Fig. 1C). However, their peptide-binding sites exhibited partial divergence, most notably the substitution at position 75 in the α1 domain of MHC-IIα, where the conserved asparagine residue was replaced (Fig. 1B). In contrast, the peptide-binding residues in the β1 domain of MHC-IIβ remained conserved, retaining proline and asparagine as in DAB molecules (Fig. S1).

Membrane protein extraction followed by MS analysis was performed to characterize the expression profiles of these MHC-II genes across different tissues. Among the annotated MHC-II genes, nine (four MHC-IIα and five MHC-IIβ) were confirmed to produce detectable protein products, all located on chromosomes A8 and B3 (Table 1 and Fig. 1A). Notably, DDA and DFA, as well as DDB and DFB, shared extremely high amino acid sequence similarity (up to 99%), making it impossible to distinguish their corresponding peptides by MS (data not shown). Nevertheless, DDA/DFA and DDB/DFB, together with the classical DAA and DAB molecules, exhibited the highest expression levels. Tissue-specific analysis showed that the primary immune organs in teleosts—the spleen and head kidney—displayed the greatest diversity and abundance of MHC-II protein expression. Moderate expression was observed in the intestine, trunk kidney, and gills, while the liver and brain exhibited minimal expression (Table 1).

**TABLE 1:** The distribution of membrane MHC-II molecules^*a*^

Gene name	Brain	Intestine	Gill	Liver	Spleen	Head kidney	Trunk kidney	Σ(# PSMs)/gene	Chr.
*daa*	2	5	4	2	6	5	3	27	A8
*dab*	0	2	1	0	2	3	1	9	A8
*ddb/dfb*	0	2	2	1	3	4	1	13	B3
*dda/dfa*	0	4	0	0	7	5	3	19	B3
*dgb*	0	0	0	0	0	1	0	1	B3
*dha*	0	0	0	0	3	1	1	5	B3
*dhb*	0	1	1	0	1	1	0	4	B3
Σ(# PSMs)/organ	2	14	8	3	22	20	9		

^*a*^ PSMs, peptide spectrum matches.

To further validate these findings, a pAb was generated against *Cagi*-DDA/DFA, the most abundantly expressed MHC-II molecule encoded on chromosome B3. Western blot analysis using membrane proteins from different tissues revealed a distinct band at ~28 kDa, consistent with the predicted molecular weight of *Cagi*-DDA/DFA (Fig. 1D). Moreover, the tissue-specific expression pattern observed by Western blotting closely corresponded with the proteomic results, confirming the reliability of the MS-based analysis.

### Analysis of *Cagi*-DDA/DFA polymorphism and allelic distribution in the population

**Fig 2:**
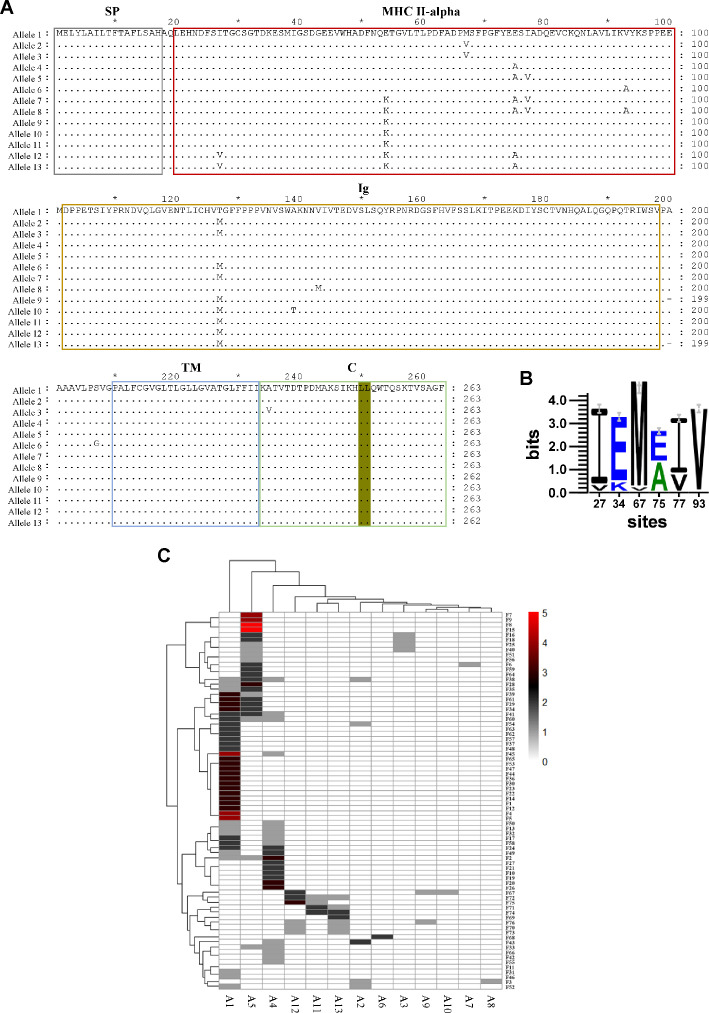
*Cagi*-DDA/DFA polymorphism and allelic distribution. (**A**) Multiple sequence alignment for *Cagi*-DDA/DFA alleles. Olive: endosomal sorting motifs. (**B**) The amino acid composition and frequency distribution of six variable sites among the 218 sequences. (**C**) The heatmap shows the allelic distribution of *Cagi*-DDA/DFA in the 76 *C*. *gibelio* population.

In teleost fish, MHC-II molecules are not only multigenic but also display extensive polymorphism. To assess the genetic diversity of *Cagi*-DDA/DFA within the *C. gibelio* population, the *Cagi*-DDA/DFA gene was amplified from 76 individuals using PCR. A total of 218 full-length sequences were obtained by randomly selecting monoclonal colonies for sequencing, which were classified into 13 unique protein isoforms (Fig. 2A). Sequence alignment revealed notable polymorphisms within the α1 domain of *Cagi*-DDA/DFA, with six amino acid substitution sites identified (Fig. 2A). The amino acid composition and frequency distribution at these six variable sites among the 218 sequences are illustrated in Fig. 2B. Among all identified alleles, allele 1 exhibited the highest frequency, accounting for 41.3% of all sequenced clones, indicating that it is the predominant allele in the *C. gibelio* population (Fig. 2C).

### Evaluation of MHC-II complex enrichment using anti-*Cagi*-DDA/DFA pAb

**Fig 3:**
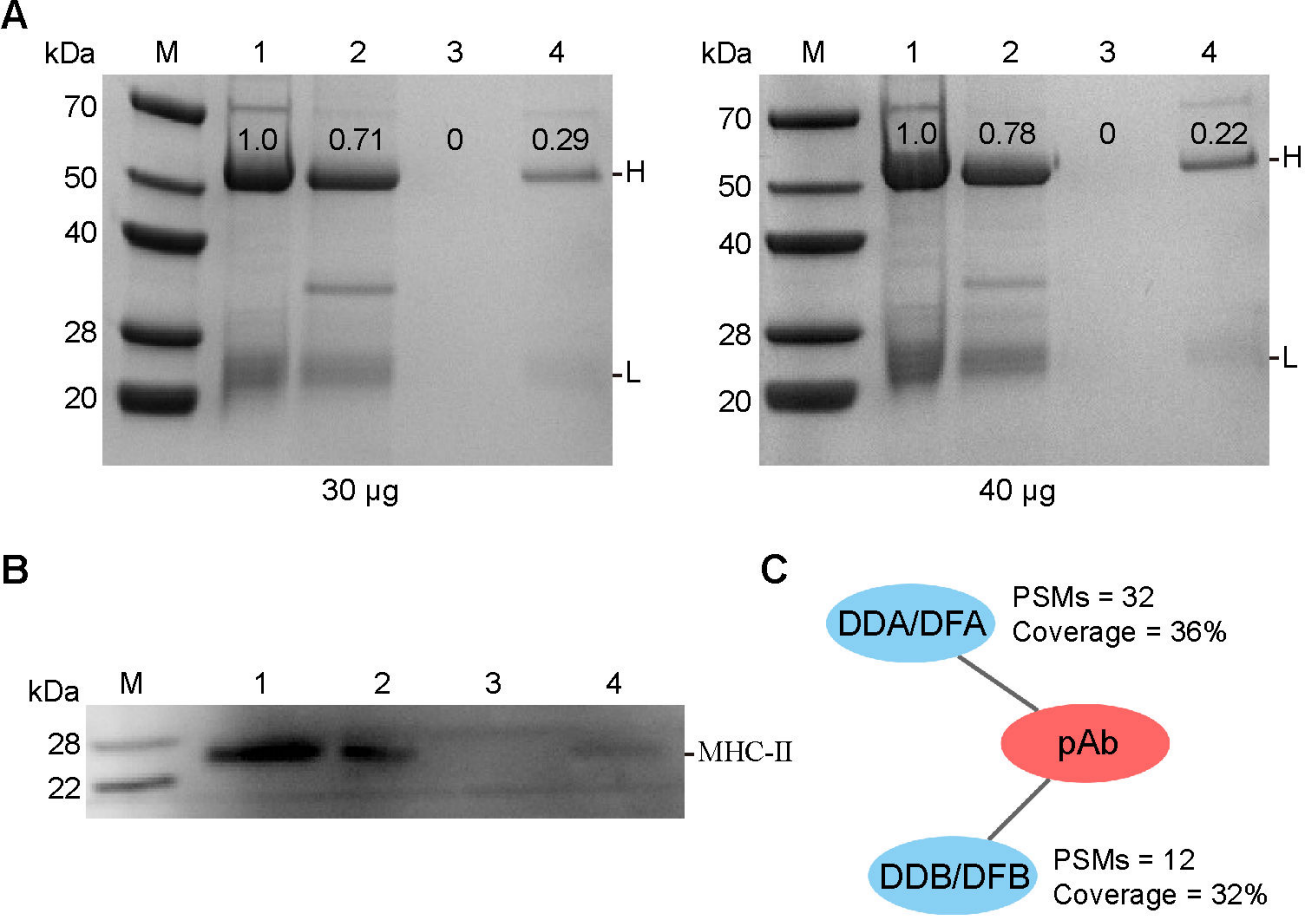
Assessment of the capture efficiency of MHC-II complexes by anti-*Cagi*-DDA/DFA pAb. (**A**) Coomassie gel staining to track different content of pAb binding efficiency to Protein A magnetic beads. Lane 1: pAb pre-coupling; Lane 2: beads coupled with pAb; Lane 3: supernatant following last wash after coupling; and Lane 4: unbound pAb following coupling step. (**B**) Western blot analysis confirming the enrichment of *Cagi*-DDA/DFA from head kidney tissue lysates via immunoaffinity purification. Lane 1: total protein lysate from head kidney tissue; Lane 2: acidic elution from antibody-conjugated magnetic beads after enrichment; Lane 3: final wash supernatant following immunoaffinity purification; and Lane 4: residual lysate after enrichment. (**C**) Identification of MHC-II complexes eluted from the pAb-conjugated magnetic beads using mass spectrometry.

An anti-*Cagi*-DDA/DFA allele 1 pAb was generated, and its specificity for *Cagi*-DDA/DFA recognition was evaluated by testing cross-reactivity with other MHC-II molecules. Western blot analysis demonstrated that the anti-*Cagi*-DDA/DFA antibodies specifically recognized recombinant DDA/DFA-flag protein, but did not bind to other recombinant MHC-II proteins such as DAA-flag, DBA-flag, and DEA-flag (Fig. S2). To further assess the immunoaffinity enrichment efficiency of MHC-II complexes using pAb against *Cagi*-DDA/DFA, a series of experiments were conducted at RT. Specifically, 30 or 40 μg of polyclonal anti-*Cagi*-DDA/DFA antibodies were conjugated to Protein A magnetic beads. Coupling efficiency was evaluated by collecting key fractions and analyzing them via SDS-PAGE followed by Coomassie Brilliant Blue staining. The results indicated that up to 70% of the antibodies successfully bound to the beads, confirming effective antibody immobilization (Fig. 3A). Subsequently, 50 μg of total protein extracted from *C. gibelio* head kidney tissue was subjected to immunoaffinity purification using the antibody-conjugated beads. Western blot analysis revealed a marked reduction of *Cagi*-DDA/DFA protein in the post-enrichment lysate, while a substantial amount of *Cagi*-DDA/DFA was detected in the acidic elution from the magnetic beads, indicating successful enrichment (Fig. 3B). Furthermore, MS analysis of the eluates confirmed the presence of *Cagi*-DDA/DFA, as well as its interacting partner, the MHC-IIβ chain (*Cagi*-DDB/DFB), suggesting that the prepared pAb effectively captured intact and functional MHC-II complexes (Fig. 3C).

### Immunopeptidome profiling of CyHV-2 MHC-II peptides

**Fig 4:**
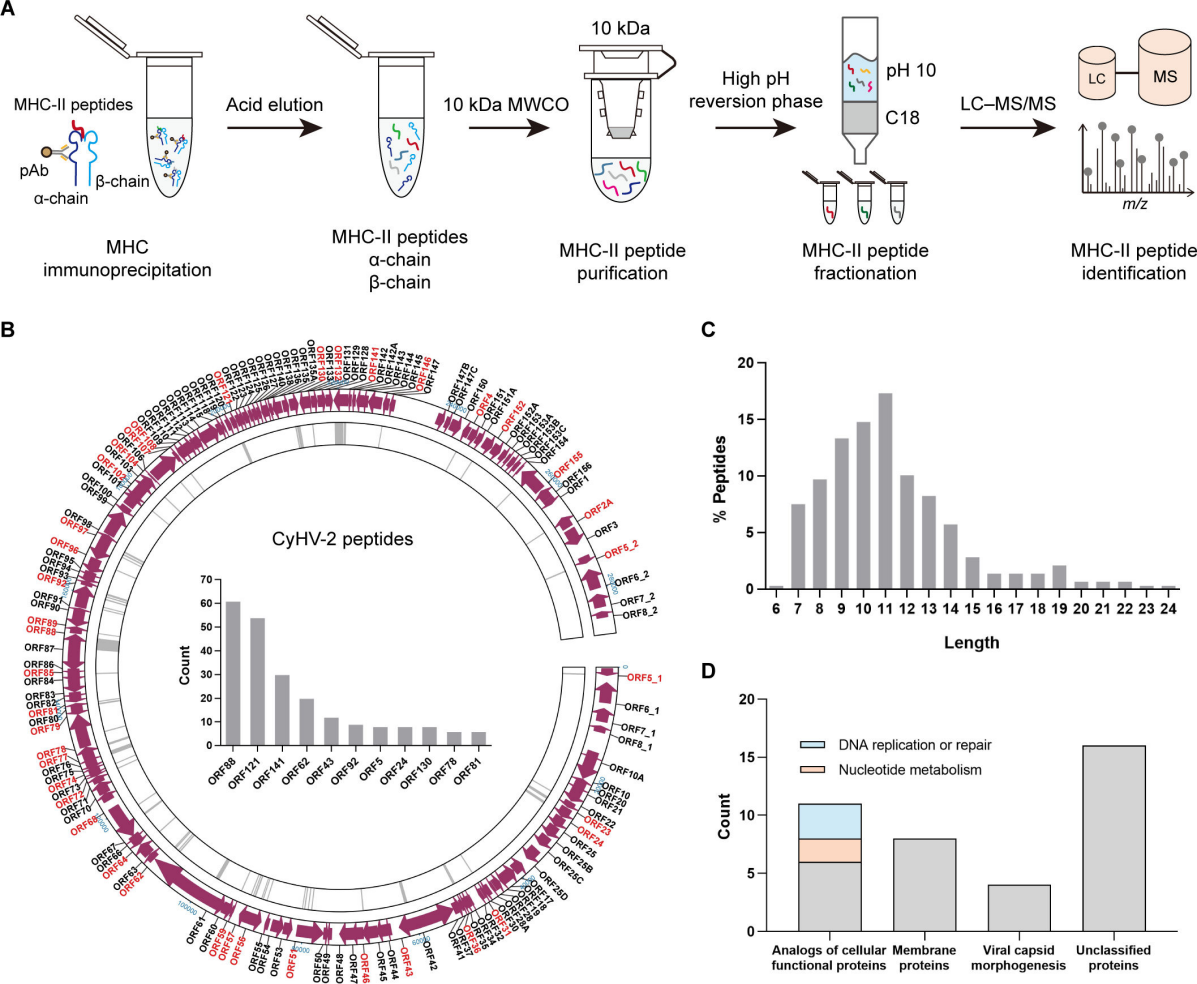
Identification of CyHV-2 MHC-II peptides. (**A**) Workflow for *C. gibelio* MHC-II-associated peptide purification, fractionation, and identification by LC-MS/MS. (**B**) Summary of peptide location across the CyHV-2 genome from the *Cagi*-DFA immunopeptidome. The CyHV-2 proteins with immunodominant antigen peptides were marked in red. The histogram in the middle of the picture shows the top 10 viral proteins that have the most identified antigen peptides. (**C**) The frequency distribution of the peptide lengths of *C. gibelio* MHC-II peptides. (**D**) Functional classification of 39 viral proteins with immunodominant antigen peptides.

Head kidney tissue from CyHV-2 infected *C. gibelio* was collected for analysis of biochemically purified *Cagi*-DDA/DFA-bound peptides. MHC-II complexes were immunoprecipitated using an anti-*Cagi*-DDA/DFA pAb, followed by peptide elution with 10% acetic acid. The eluted peptides were then purified using a 10 kDa molecular weight cutoff ultrafiltration device. To reduce sample complexity, the peptide mixture was fractionated using high-pH reversed-phase chromatography and subsequently subjected to MS analysis (Fig. 4A). In total, 276 unique peptides derived from 39 viral proteins were identified in the MHC-II peptidome of *C. gibelio* (Table S1). The genomic distribution of these CyHV-2-derived peptides is shown in Fig. 4B. Most peptides were 9–12 amino acids in length (Fig. 4C). Among the identified viral proteins, CyHV-2 ORF88, ORF121, and ORF141 yielded the highest number of MHC-II peptides, suggesting their strong antigenic potential (Fig. 4B). And many peptides were also derived from the viral major capsid protein (CyHV-2 ORF92), a known immunodominant antigen, reinforcing its role as a primary target recognized by the host adaptive immune system.

To further characterize the antigenic landscape of CyHV-2, the 39 source viral proteins were classified based on their predicted biological functions. After excluding 16 proteins with unknown functions or lacking defined structural domains, the remaining 23 proteins were grouped into three major categories: (i) analogs of cellular functional proteins (ii), membrane proteins, and (iii) proteins involved in viral capsid morphogenesis (Fig. 4D). Among the analogs of cellular proteins, nearly half were predicted to participate in nucleotide metabolism and DNA replication or repair. These findings provide a comprehensive overview of the antigenic properties of CyHV-2 proteins and offer valuable insights for the selection of potential vaccine targets.

### Highly abundant and early-expressed proteins of CyHV-2 are key immunodominant antigens

**Fig 5:**
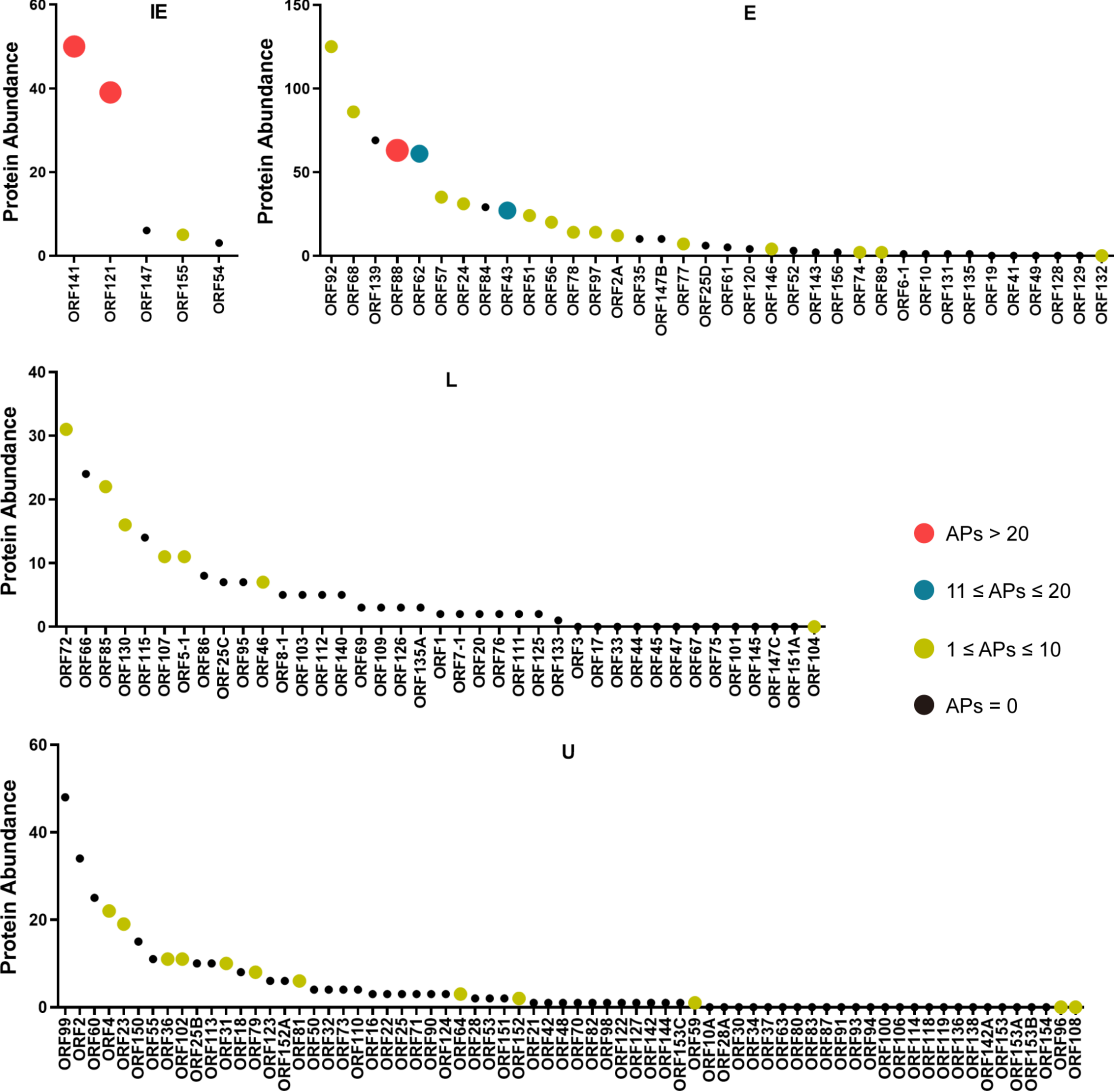
Correlation between MHC-II antigen presentation and viral protein abundance and temporal expression. The 39 CyHV-2 proteins identified with MHC-II peptides were classified into four temporal expression categories: immediate-early (IE), early (E), late (L), and undefined (U). Proteins were color-coded based on the number of antigen peptides (APs) identified. Protein abundance was estimated using peptide-spectrum matches (PSMs) from our previously published proteomic data set (28).

Similar to other DNA viruses, CyHV-2 genes are categorized into three temporal classes: immediate-early (IE), early (E), and late (L) genes (31). By integrating these published data sets with our findings, we observed that the 39 viral proteins identified in this study as having significant antigenicity tend to be highly expressed and are rapidly transcribed during early stages of infection (Fig. 5). First, the immunodominant viral proteins are predominantly enriched in the highly expressed fractions across all gene expression categories. Moreover, the proportion of antigenic proteins is higher among IE and E proteins compared to L proteins. In terms of MHC-II peptide yield, IE and E proteins also contributed more peptides than L proteins. For instance, among the five currently known IE proteins, three were found to possess notable antigenicity, exhibiting both high expression levels and high numbers of identified MHC-II peptides (Fig. 5). These findings suggest that CyHV-2 proteins with high abundance and early expression are more likely to elicit adaptive immune responses in the host, making them promising targets for future vaccine development.

## DISCUSSION

In this study, we systematically analyzed the genomic distribution and tissue-specific expression of MHC-II genes in *C. gibelio*. Chromosome B3 was identified as the major locus for MHC-II gene localization and expression, with *Cagi*-DDA/DFA showing the highest expression level, suggesting its pivotal role in antigen presentation. Furthermore, by combining immunoaffinity enrichment with MS, we successfully identified CyHV-2-derived antigen peptides associated with *Cagi*-DDA/DFA. These results demonstrate the feasibility of profiling *Cagi*-DDA/DFA-bound viral peptides and provide a theoretical basis for target selection in future vaccine development. However, the following issues remain to be addressed.

Both *Cagi*-DDA/DFA and *Cagi*-DDB/DFB are located on chromosome B3 of *C. gibelio* and exhibit nearly identical sequences, suggesting a tandem gene duplication event in this region. Since the pAb and MS-based approach used here cannot distinguish between these proteins, the specific gene cluster forming functional MHC-II complexes remains to be clarified. In addition, several identified *Cagi*-DDA/DFA-bound peptides were shorter than the typical MHC-II ligand length and may result from non-specific cleavage or fragmentation during sample preparation and MS analysis. Thus, further *in vitro* assays are required to validate the binding capability of these short peptides.

The actual expression level of *Cagi*-DDA/DFA protein on the hepatic cell membrane of *C. gibelio* was significantly lower than expected. In teleosts, the liver is not only a central metabolic organ but also plays a role in immune defense (32). This is evidenced by the frequent presence of immune cell infiltration, including mast cells, rodlet cells, neutrophils, macrophages, CD4^+^ T cells, and CD8^+^ T cells et al., within healthy or pathogens-infected hepatic tissues (33–35). Upon pathogen challenge, liver cells exhibit marked upregulation of immune-related genes (36, 37). Moreover, quantitative results have shown that the liver, along with the spleen and kidney, serves as a major site of CyHV-2 accumulation (38). Despite this, our findings revealed that the expression of MHC-II proteins in the liver was comparable to that in the brain, which is generally considered devoid of MHC-II expression and markedly lower than in other immune organs. A similar pattern has also been observed in Sichuan taimen (*Hucho bleekeri*), in which the mRNA levels of both MHC-IIα and MHC-IIβ in the liver were significantly lower than those in other immune organs (39). In contrast, MHC-I molecules are typically expressed at relatively high levels in the teleost liver and are significantly upregulated following viral infection (40–42). These observations suggest that the liver in teleost fish primarily contributes to immune defense through innate immunity or MHC-I-mediated CD8^+^ T cell responses, whereas MHC-II-mediated antigen presentation plays a comparatively minor role in this organ.

Viral proteins that are expressed early and at high abundance during CyHV-2 infection exhibit enhanced antigenicity. Previous studies in humans have reported that highly abundant and rapidly turned-over proteins are more likely to generate peptides capable of binding to HLA-I molecules (43). This may be attributed to their increased likelihood of being recognized and degraded by the host, leading to the generation of a larger pool of peptides with greater chances of binding intracellular MHC molecules. A similar pattern was observed in *C. gibelio* infected with CyHV-2, where viral proteins with high abundance or early expression tended to contain multiple MHC-II peptides. Viral IE genes are transcribed immediately upon viral entry, without requiring prior synthesis of viral proteins (31, 44). This enables IE proteins to be among the first recognized by the host immune system. Their elevated expression levels—potentially essential for establishing a cellular environment conducive to viral replication—may further contribute to their immunogenicity. Therefore, early and abundant viral proteins represent promising targets for pathogen detection and antiviral drug development.

MHC-II-bound peptides can be co-administered with corresponding antigens to enhance CD4^+^ T cell activation, thereby boosting the overall immunogenicity of the target antigen (45). Although MHC-II peptides alone hardly directly induce neutralizing antibodies or antigen-specific CD8^+^ T cell responses, the elicited CD4^+^ T cells play a central regulatory role in both cellular and humoral immunity by promoting the maturation and differentiation of CD8^+^ T cells and B cells (46). For example, coupling target antigens with pan HLA-DR-binding epitopes (PADRE) has been widely employed to optimize CD4^+^ T cell responses and improve the protective efficacy of subunit vaccines (47–49). Therefore, leveraging the identified MHC-II-bound CyHV-2 peptides for the rational design and development of diverse vaccine formulations represents a promising direction for future antiviral vaccine research.

In conclusion, this study identified CyHV-2-derived peptides bound to MHC-II molecules in *C. gibelio*, providing a valuable data set of CD4^+^ T cells antigenic epitopes. These findings shed new light on the interaction between the teleost adaptive immune system and viral pathogens and provide important insights for future vaccine development.

## Data Availability

The mass spectrometry proteomics data have been deposited to the ProteomeXchange Consortium via the iProX partner repository (ProteomeXchange: PXD066128; iProX: IPX0012573001), which are publicly accessible at http://proteomecentral.proteomexchange.org and https://www.iprox.org, respectively.

## SUPPLEMENTAL MATERIAL

Fig. S1 jvi.01870-25-s0001.tif Alignments of β1 domain sequences.

Fig. S2 jvi.01870-25-s0002.tif Evaluation of anti-*Cagi*-DDA/DFA pAb specificity.

Supplemental legends jvi.01870-25-s0003.docx Legends for Fig. S1 and S2.

Table S1 jvi.01870-25-s0004.xlsx *C. gibelio* MHC-II-bound CyHV-2 peptides.

ASM does not own the copyrights to Supplemental Material that may be linked to, or accessed through, an article. The authors have granted ASM a non-exclusive, world-wide license to publish the Supplemental Material files. Please contact the corresponding author directly for reuse.
