# Supplementary figures and images for: Analysis of MHC class II-bound CyHV-2 peptides in Carassius gibelio using mass spectrometry
Source: J Virol. 2025 Dec 29;100(2):e01870-25. doi: 10.1128/jvi.01870-25 (PMC12911862; doi:10.1128/jvi.01870-25)

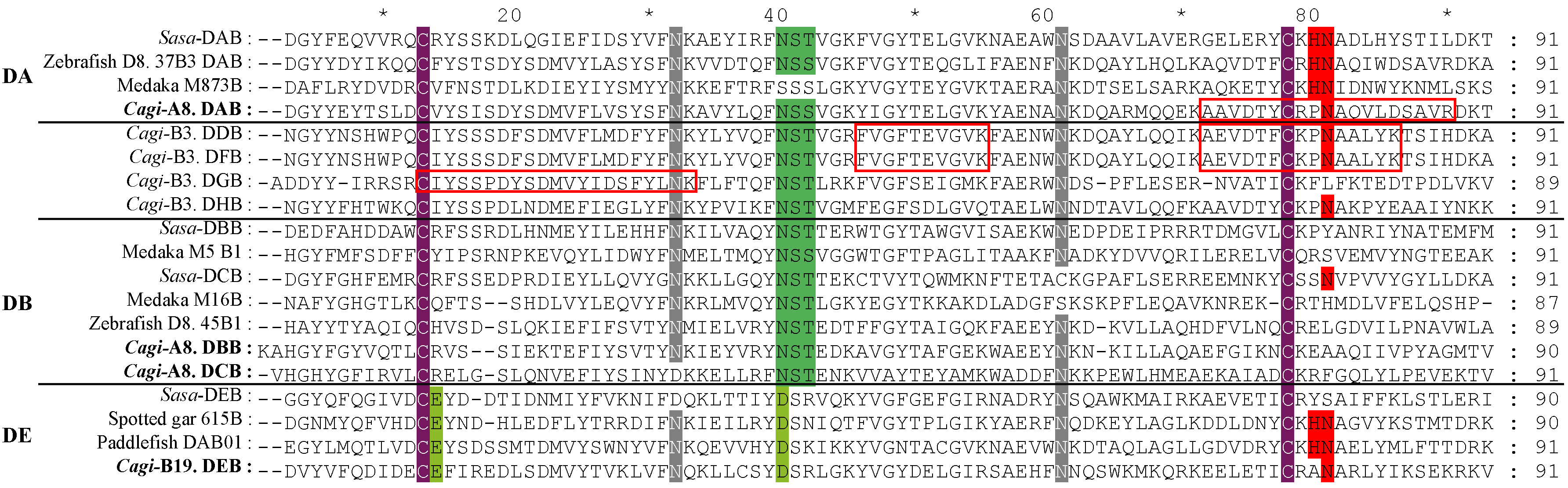

Supplement: Fig. S1 — Alignments of β1 domain sequences. [file jvi.01870-25-s0001.tif]

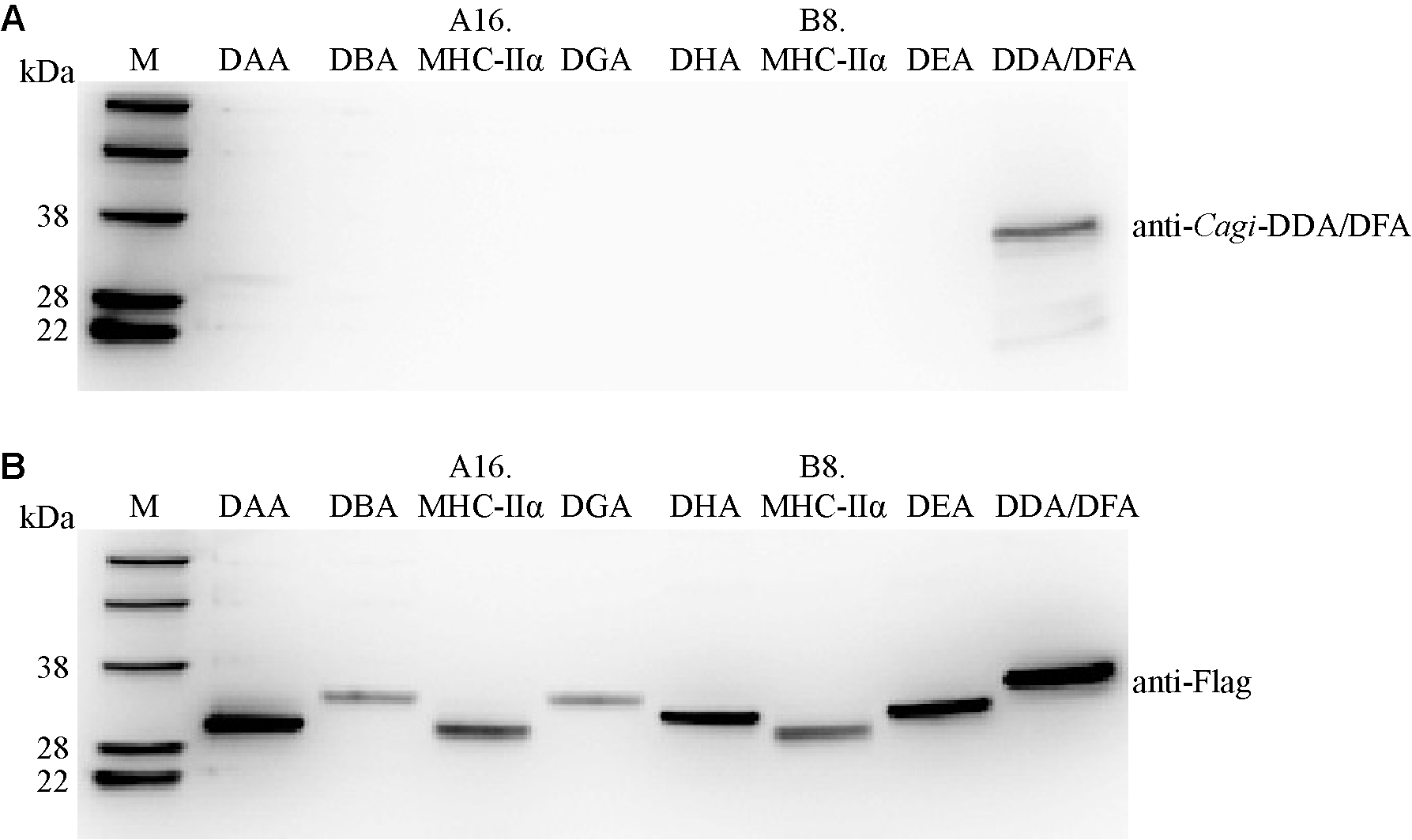

Supplement: Fig. S2 — Evaluation of anti-Cagi-DDA/DFA pAb specificity. [file jvi.01870-25-s0002.tif]
