# Supplementary material for: Analysis of MHC class II-bound CyHV-2 peptides in Carassius gibelio using mass spectrometry
Source: J Virol. 2025 Dec 29;100(2):e01870-25. doi: 10.1128/jvi.01870-25 (PMC12911862; doi:10.1128/jvi.01870-25)
Supplement: Supplemental legends — Legends for Fig. S1 and S2. [file jvi.01870-25-s0003.docx]

**Supporting information:**

**Figure S1: Alignments of β1 domain sequences.** Representative teleost fish DAB, DBB and DEB group sequences are compared with *C. gibelio* MHC-Ⅱβ molecules. Purple: cysteine residues predicted to form disulfide bridges; Red: peptide-backbone interacting residues; Lime green: DE group specific residues; Green: conserved N-glycosylation motifs; Gray: highly conserved residues. Red frame indicates peptides identified by MS.

**Figure S2: Evaluation of anti-*Cagi*-DDA/DFA pAb specificity.** (A) Western blot analysis of various recombinant MHC-Ⅱ-Flag proteins using the anti-*Cagi*-DDA/DFA pAb. (B) Parallel Western blot analysis of the same protein series probed with an anti-Flag antibody to confirm protein expression. 50 µL total protein extracts from transfected GiCB cells were loaded in each lane.
